# Duration of anti-seizure medicines started for acute symptomatic seizures due to acute meningitis: a systematic review and meta-analysis

**DOI:** 10.1186/s12916-026-04840-w

**Published:** 2026-04-22

**Authors:** Manya Prasad, Amit Kumar, Rachel Couban, Nicoline Schiess, Kavita Kothari, Elaine Brohan, Nicolo Binello, Francesco Venuti, Tarun Dua

**Affiliations:** 1 Centre for Community Medicine, All India Institute of Medical Sciences (AIIMS), New Delhi, India; 2 Rajendra Institute of Medical Sciences (RIMS), Ranchi, India; 3 McMaster University, Hamilton, Canada; 4 Department of Mental Health, Brain Health Unit, Brain Health and Substance Use, World Health Organization, Geneva, Switzerland; 5 Consultant to Library & Digital Information Networks, World Health Organization, Geneva, Switzerland; 6 Emerging Zoonoses and High Impact Epidemics, World Health Organization (WHO), Geneva, Switzerland

**Keywords:** Anti-seizure medicines, Meningitis, Sequelae, Meta-analysis

## Abstract

**Background:**

Acute symptomatic seizures (ASS), a frequent complication of meningitis, are potentially life-threatening and are a predictor of epilepsy if not managed appropriately. The optimal duration of anti-seizure medicines (ASM) in these patients remains unclear. This systematic review evaluates the evidence for earlier versus later cessation of ASM in patients with meningitis who experience ASS.

**Methods:**

A comprehensive literature search was conducted across Medline via OVID, Embase via OVID, Cochrane CENTRAL, Web of Science, and ClinicalTrials.gov. Eligible studies included randomized controlled trials and cohort studies that compared ASM cessation within 3 months of initiation of treatment to cessation beyond 3 months. Pooled estimates for outcomes including epilepsy development, seizure recurrence, and adverse events were calculated using random-effects meta-analysis. The certainty of evidence was assessed using the GRADE methodology.

**Results:**

Out of 4283 records screened for eligibility, none were found to provide direct evidence in people with meningitis. Two studies on people with encephalitis were included in the meta-analysis as indirect evidence. A randomized controlled trial (RCT) compared 4 weeks versus 12 weeks of ASM in children with acute encephalitis syndrome, while a cohort study investigated ASM duration in adults, including a subset with bacterial meningitis. Both studies found no significant difference in seizure recurrence between earlier and later cessation groups. The pooled risk ratio for seizure recurrence was 1.14 (95% CI, 0.26–5.01). The certainty of the evidence was very low due to indirectness and imprecision. Indirect evidence from other causes of ASS was inconclusive with regard to the duration of ASM.

**Conclusions:**

The available evidence is inconclusive with regard to the difference between early and late ASM cessation in reducing seizure recurrence. Due to very low certainty of evidence, individualized clinical judgment remains crucial. Further research, specifically targeting bacterial meningitis, is needed to clarify optimal ASM duration in this population.

**Supplementary Information:**

The online version contains supplementary material available at 10.1186/s12916-026-04840-w.

## Background

Acute symptomatic seizures (ASS) frequently occur as a complication of acute meningitis, requiring changes in clinical management [1]. Studies show that acute symptomatic seizures (ASS) occur in approximately 17–27% of adults with bacterial meningitis [2]. Anti-seizure medicines (ASM) are commonly prescribed to control seizures and prevent recurrent episodes, but the optimal duration of their use remains unclear.

ASS in the context of meningitis pose unique challenges to healthcare providers. The decision of whether to initiate and when to discontinue ASM is of great clinical importance. Inappropriate and prolonged use of ASM may expose patients to unnecessary side effects, drug interactions, and increased healthcare costs. Conversely, premature discontinuation of these medications can lead to recurrent seizures, which may lead to adverse effects on the brain with short-term and long-term medical, social, and economic consequences.

To date, there is no comprehensive synthesis of existing evidence to guide clinical practice in people with ASS from acute meningitis. This systematic review was designed to address the unresolved question of the optimal duration of ASM administration in people with acute bacterial meningitis who have experienced acute symptomatic seizures.

## Methods

The protocol for this systematic review was published on PROSPERO (CRD42023484944).

### PICO question

What should be the duration of anti-seizure medicines in individuals with acute meningitis who were started on this treatment for acute symptomatic seizures?

### Criteria for selection

#### Population (P)

Adults and children with acute meningitis experiencing acute symptomatic seizures and receiving anti-seizure medicines.

#### Intervention (I)

Early stopping of anti-seizure medicines (within three months of the administration of medicines).

#### Comparison (C)

Late stopping (beyond three months) of anti-seizure medicines.

#### Outcomes (O)

##### Critical outcomes

- Development of epilepsy (recurrence of two or more unprovoked seizures)
- Adverse effects of medicines.
- Mortality

##### Important outcome

Recurrence of seizure.

### Study designs

1. Experimental and quasi-experimental studies
2. Randomized controlled trials
3. Non-randomized studies of intervention
4. Observational studies
5. Cohort studies (retrospective, non-concurrent, and prospective)
6. Case-series

Studies should have estimated the differences in the outcomes between the groups receiving the intervention of interest or the comparator.

### Published language

The intention was to include studies published in all languages.

### Exclusion criteria

- Preclinical studies (in vivo and in vitro studies)
- Studies without a control group
- Records of registered, ongoing trials with no results (e.g., those from clinicaltrials.gov)
- The following disease categories were excluded: meningitis in newborns (0–28 days); hospital-acquired, nosocomial and health-care-associated meningitis; subacute and chronic meningitis, including tuberculous, cryptococcal and eosinophilic meningitis; non-infectious meningitis (e.g., drugs, malignancy, autoimmune diseases).

### Search strategy

#### Information sources

The following databases were searched: Medline via OVID, Embase via OVID, Cochrane CENTRAL, Web of Science, and Clinicaltrials.gov on 19th December 2023. Reference lists of all included studies and relevant reviews were reviewed for additional references. The search used the concepts of meningitis and anticonvulsants. Limits were used to remove animal studies and ineligible records such as case reports, comments, editorials, letters and reviews (Appendix I).

#### First stage

Two reviewers independently screened titles and abstracts to determine studies eligible for full-text screening. Disagreements were resolved by discussion or by referring to a third reviewer.

#### Second stage

Two reviewers independently reviewed the full texts of potentially eligible studies to determine the final eligible studies. Disagreements were resolved by discussion or by referring to a third reviewer.

Rayyan software was used for screening of titles/abstracts and full text articles [3]. The reference lists of the eligible articles were retrieved and screened. Moreover, a subject expert was consulted to identify further eligible articles.

### Data extraction and management

The data was extracted using a pilot-tested standardized data collection template. Two of the authors independently extracted data from the eligible records. In case of disagreement, consensus was obtained through discussions. In case of persistent disagreement, the opinion of a third author was considered binding. The following information was extracted from each manuscript: surname of the first author; year of publication; country; region; sample size; enrolment period; details on population (etiology, mean age, % male, disease severity, type of treatment received before or during therapy); interventions (anti-seizure drug, dose, duration, route); length of follow-up; outcomes reported; and effect sizes with 95% CIs.

### Assessment of risk of bias in studies included in the review

The risk of bias in randomized trials was assessed using the Cochrane Risk of Bias 2 for randomized controlled trials, ROBINS-I for non-randomized studies, and Joanna Briggs Institute (JBI) checklist for case series [4–6]. Two reviewers independently assessed the study risk of bias, with disagreements resolved by involving a third reviewer.

### Data synthesis

If there was a consistent outcome measure across two or more studies, meta-analyses for the effect estimate of the interventions were performed. Pooled odds ratio (OR), relative risk (RR), and 95% confidence intervals (95% CI) were calculated for dichotomous outcomes with mean difference (MD) or standardized mean difference (SMD) and 95% CI for continuous outcomes.

### Assessment of certainty of evidence (GRADE evidence profiles)

Grading of Recommendation, Assessment, Development, and Evaluation (GRADE) methodology was used to rate the certainty of evidence for each outcome as high, moderate, low, or very low [7]. The assessment included judgments addressing risk of bias, imprecision, inconsistency, indirectness, and publication bias. Evidence was summarized both narratively and in GRADE evidence profiles using GRADEpro software [8].

Two of the authors independently assessed the certainty of evidence for the synthesized estimates. In case of disagreement, consensus was obtained through discussion. In case of persistent disagreement, the opinion of a third reviewer was considered binding. A minimally contextualized framework within the GRADE framework was used to assist guideline development. Our target of certainty rating was a non-null effect.

### Analysis of subgroups or subsets and investigation of heterogeneity

Heterogeneity in the meta-analyses was assessed by visual inspection of the forest plot and by the *I*^2^ statistic. A threshold of 50% was considered for assessment of heterogeneity using the *I*^2^ statistic.

### Deviations from the review protocol

In the absence of direct evidence from acute bacterial meningitis, evidence from people with ASS due to other causes, such as acute encephalitis syndrome, was included. It was noted that seizures in meningitis are an indication of parenchymatous brain inflammation, but the severity and extent of inflammation of brain parenchyma in encephalitis may be more than in meningitis, and hence seizures in encephalitis may be more refractory or have a higher tendency to recur than in meningitis. Using this rationalization, if a shorter duration of treatment was as effective as a longer duration in encephalitis, it was likely also to be effective in meningitis. Thus, consideration of indirect evidence from encephalitis served as a “worst-case” scenario for meningitis.

Additionally, evidence for key considerations such as the absolute risk of seizure recurrence in people with ASS post-acute meningitis was also narratively synthesized. This was undertaken to contextualize decisions on ASM continuation or withdrawal, as the clinical trade-off between prolonged treatment and early cessation is strongly influenced by the underlying absolute risk of recurrent seizures, which may be higher or lower than commonly assumed by clinicians.

## Results

### Search results

**Fig. 1:**
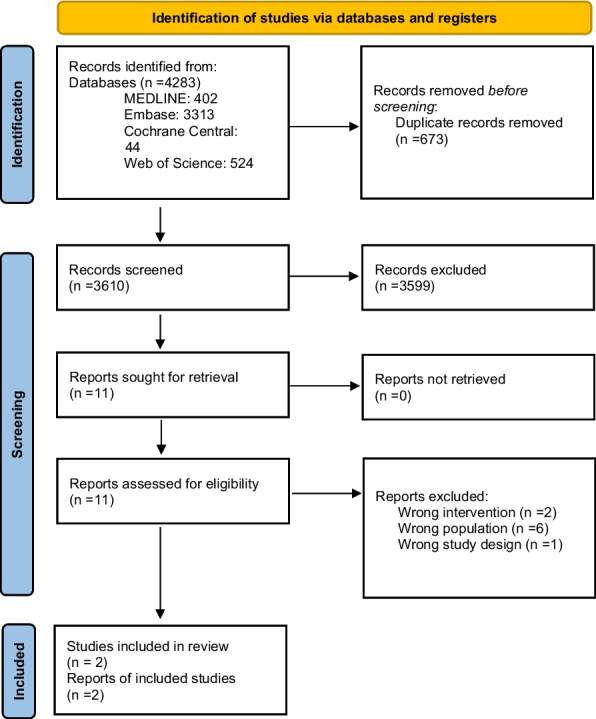
PRISMA flow diagram for the systematic review

Our search yielded 4283 titles and abstracts—all identified from the electronic database search, 3610 remained after duplicates were removed, and 3599 articles were excluded based on a review of the title and abstract, leaving 11 articles for full review. Of these, eight were excluded for the following reasons: wrong study design (*n* = 1), wrong intervention (*n* = 2), wrong population (*n* = 6). Two studies were included in the systematic review. Figure 1 provides the review PRISMA flow diagram.

### Included studies

Two studies met the inclusion criteria (Table 1):

1. Dhawan et al. (2021) [9]—A randomized controlled trial comparing 4 weeks versus 12 weeks of ASM in children with acute encephalitis syndrome. The study enrolled 60 participants (30 in each arm) and measured seizure recurrence at 6-, 12-, and 18-month post-intervention. The majority of children had aseptic meningitis/meningoencephalitis (*n* = 29, 28.3%).
2. Herzig-Nichtweiß et al. (2023) [10]—A prospective multicenter observational cohort study investigating the management of ASS, including a subset of patients with bacterial meningoencephalitis or infectious meningitis (7%). The study compared seizure recurrence in patients receiving ASM for less than 100 days versus more than 100 days, with a follow-up of 12 months. A total of 120 participants were enrolled, with 53 in the early stopping group and 67 in the late stopping group.

**Table 1:** Characteristics of studies included in the GRADE evidence profile

Study name, year, country	Study design	Overall risk of bias (study level)	Other key detail of intervention	Population (sample size: Intervention/control)	Comparator	Outcome domains with available data (synthesis method/metric)	Specific outcomes measure	Time point of measurement
Studies including patients of acute encephalitis/meningoencephalitis								
Dhawan S R, 2021, India [7]	RCT	Low	4 weeks anti-seizure medicines	Acute encephalitis syndrome The majority of children had aseptic meningitis/meningoencephalitis (*n* = 29, 28.3%) 30/30	12 weeks anti-seizure medicines	Seizure recurrence, adverse effects	Seizure recurrence	6, 12, and 18 months
Herzig‐Nichtweiß 2023 [8]	Cohort study	Low	ASM less than 100 days	Adults 8% bacterial meningoencephalitis or meningitis; cerebrovascular accidents formed the most prevalent group (*n* = 90; 75%), followed by infections with structural affection of brain tissue visible on neuroimaging (*n* = 14; 11%) (53/67)	ASM more than 100 days	Seizure recurrence	Seizure recurrence	12 months

Population: Adults and children with acute symptomatic seizures with acute meningitis receiving anti-seizure medications

Intervention: Early stopping of antiseizure medication

Comparator: Late stopping of anti-seizure medication

### Risk of bias assessment

Dhawan et al. [9] were assessed as having low risk of bias in most domains. The randomization process was adequately conducted using a computer-generated allocation sequence, allocation concealment, and baseline characteristics were well-balanced between groups. The study did have some concerns regarding deviations from the intended intervention due to its open-label design, which could have influenced patient management. However, outcome assessments were blinded, and there was no significant loss to follow-up, ensuring a low risk of bias in outcome measurement and reporting (Appendix II).

Herzig-Nichtweiß et al. [10] were rated as having low risk of bias across most domains, including confounding, selection bias, and reporting bias. However, there was a potential high risk of bias related to deviations from the intended intervention. The study demonstrated low risk of bias in terms of missing data and outcome measurement (Appendix II).

### Outcomes measured

#### Seizure recurrence

The critical outcome seizure recurrence was reported in both studies. The randomized controlled trial [9] was inconclusive with regard to seizure recurrence between the 4-week and 12-week groups at 12 months, with an identical recurrence rate of 3.3% in both groups (risk ratio (RR) 1.00; 95% CI, 0.06 to 16.68). The cohort study [10] similarly reported an inconclusive estimate for seizure recurrence between the earlier cessation (less than 100 days) and later cessation (more than 100 days) groups, with a relative risk of 1.20 (95% CI, 0.21 to 6.84).

#### Adverse events

Only Dhawan et al. [9] study assessed adverse events, reporting no adverse events in either group throughout the trial.

#### Pooled effect and overall certainty

**Fig. 2:**
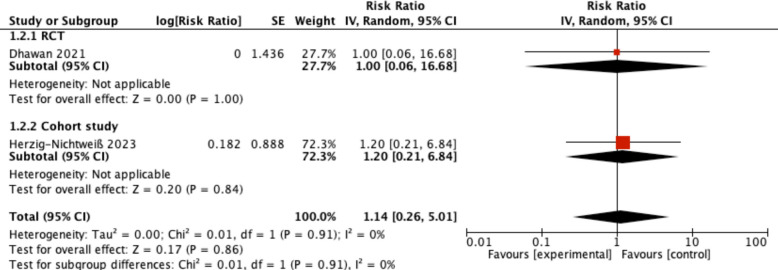
Seizure recurrence at 12 months

Very low certainty evidence from two studies was inconclusive with regard to the effect of early stopping of ASM on seizure recurrence (RR of 1.14 (95% CI, 0.26 to 5.01) (Fig. 2 and Table 2). The certainty of evidence was rated down for indirectness and imprecision.

**Table 2:** GRADE evidence profile

Certainty assessment							Summary of findings				
**Participants (studies) Follow-up**	**Risk of bias**	**Inconsistency**	**Indirectness**	**Imprecision**	**Publication bias**	**Overall certainty of evidence**	**Study event rates (%)**		**Relative effect (95% CI)**	**Anticipated absolute effects**	
							**With late stopping of ASM**	**With early stopping**		**Risk with late stopping of ASM**	**Risk difference with Early stopping**
**Seizure recurrence at 12 months–RCT**											
60 (1 RCT)	Not serious	Not applicable	Serious^1^	Very serious^2^	None	⨁◯◯◯ Very low	1/30 (3.3%)	1/30 (3.3%)	**RR 1.00** (0.06 to 16.68)	33 per 1000	**0 fewer per 1000** (from 31 fewer to 523 more)
**Seizure recurrence at 12 months–cohort study**											
141 (1 non-randomised study)	Not serious	Not applicable	Serious^1^	Very serious^2^	None	⨁◯◯◯ Very low	17%^3^	−/0	**RR 1.20** (0.21 to 6.84)	142 per 1,000	**34 more per 1000** (from 134 fewer to 993 more)

^1^Population includes patients of acute symptomatic seizures from causes apart from meningitis, such as acute encephalitis syndrome, HIE, stroke

^2^Confidence interval includes both important benefit and harm

^3^Baseline risk from: Zoons E, et al. Neurology. 2008 May 27;70(22 Pt 2):2109–15. https://doi.org/10.1212/01.wnl.0000288178.91614.5d

#### Risk of epilepsy after acute symptomatic seizures in bacterial meningitis

To inform clinical decision-making regarding ASM duration, we additionally synthesized evidence on the absolute risk of epilepsy following acute symptomatic seizures due to meningitis. A retrospective cohort study by Annegers et al. [11], which followed 714 survivors of meningitis and encephalitis over a 20-year period, provided critical insight into this risk. The study revealed that individuals who had experienced early seizures during bacterial meningitis had a significantly higher risk of developing epilepsy (13%) compared to those without early seizures (2.4%). This finding underscores the potential long-term neurological impact of early seizures in bacterial meningitis. However, it is noted that the absolute risk of repeat seizures in a 1-year duration was less than 5% after meningitis.

Similarly, a study by Hesdorffer et al. [12], involving 262 people with ASS due to various central nervous system (CNS) insults (including bacterial meningitis, stroke, and traumatic brain injury), demonstrated an elevated risk of subsequent unprovoked seizures in individuals with ASS. The 10-year follow-up data indicated that individuals who experienced early unprovoked seizures had a markedly higher risk of developing subsequent unprovoked seizures (64.8%) compared to those whose seizures were only acute symptomatic in nature (18.7%).

Chang et al. [13] further contributed to this body of evidence by investigating 116 children with bacterial meningitis, 55 of whom experienced seizures during the acute phase. The study showed that children with early seizures had a 22% probability of developing epilepsy later, while those without early seizures had no recorded instances of subsequent unprovoked seizures (Table 3).

**Table 3:** Studies on the risk of epilepsy in cases of acute symptomatic seizures following brain infections

Study name, year, country	Study design	Overall risk of bias (study level)	Population (sample size: intervention/control)	Patient characteristics	Comparator	Outcome	Follow up duration	Number with outcome/total
Annegers 1988 [9] USA	Retrospective cohort	Moderate	Population-based cohort of 714 survivors of encephalitis or meningitis between 1935 and 1981	Bacterial meningitis was present most commonly in children under 5	Bacterial meningitis without early seizure	Development of epilepsy (unprovoked seizure)	20 years	BM with early seizure = 13% BM without early seizure = 2.4%
Chang 2004 Taiwan [10]	Retrospective cohort study	Moderate	Patients with infantile and childhood bacterial meningitis (N-116) 55 had seizures during the acute phase of bacterial meningitis	19 febrile seizure after fever episodes*, 11 both a febrile seizure and underlying anatomic pathologies, 18 anatomic pathologies	Patients without seizures in acute phase	Subsequent unprovoked seizure	16 years	Patients with early seizure: 12/55 (22%) Patients without early seizure (0/61) (0%)

^*^May be mistaken use of the term by authors of the original article

The studies on the risk of epilepsy after acute bacterial meningitis are presented in Table 4. Edmond et al. [14] conducted a comprehensive systematic review that focused on the long-term outcomes of survivors of acute bacterial meningitis with a particular emphasis on epilepsy and other neurological sequelae. The review analyzed data from 18,183 survivors of bacterial meningitis, with a median study size of 85 survivors. The studies primarily included children under five years of age, as this group was most affected by bacterial meningitis. One of the key findings from the review was that 12.6% of individuals ultimately developed seizures or epilepsy, highlighting a considerable risk of long-term neurological complications following bacterial meningitis. The review also reported that the most common pathogens responsible for meningitis were *Haemophilus influenzae* (35.5%), *Streptococcus pneumoniae* (19.6%), and *Neisseria meningitidis* (16.4%), with these pathogens being linked to varying risks of neurological sequelae, including epilepsy. Further supporting this evidence, Grimwood et al. [15] conducted a prospective cohort study in Australia that followed children aged 3 months to 14 years who had survived bacterial meningitis. Over a follow-up period of 6.7 years, 3.9% of the children developed epilepsy, while none of the controls did. Another long-term retrospective cohort study by Roed et al. [16] examined children with *Haemophilus influenzae* meningitis and found a significantly elevated risk of epilepsy compared to age- and sex-matched controls, with a median follow-up of 21.3 years.

**Table 4:** Studies on the risk of epilepsy in meningitis

Study name, year, country	Study design	Overall risk of bias (study level)	Population (sample size: intervention/control)	Patient characteristics	Comparator	Outcome	Follow up duration	Number with outcome/total
Children								
Grimwood 1995 [11] Australia	Prospective cohort study	Moderate	Children who had bacterial meningitis when they were aged 3 months to 14 years. (130/130)	Age at admission (months in median): 18 Etiology of bacterial meningitis: H. influenzae type b: 100/1311 (76%); S. pneumoniae: 18/1311 (14%); N. meningitidis: 7/1311 (5%); other or unknown: 6/1311 (5%)	Grade- and sex-matched children with no history of meningitis	Diagnosis of epilepsy	6.7 years	BM group = 5/127 Control group = 0/129
Roed 2011 [12] Denmark	Retrospective cohort study	Moderate	Children who had H. influenzae meningitis, compared against age- and sex-matched controls (1242/7452)	H. influenzae meningitis group: Children who had H. influenzae meningitis, age of 0–5 years in the period of 1977 to 1996. Patients were identified from the Danish National Hospital Register	Control group: age- and sex-matched controls	Diagnosis of epilepsy	Median 21.3 years (IQR, 17–26 years)	Diagnosis of epilepsy (inpatient admission rates for epilepsies/seizure disorders; 20– < 25: 2.61 (1.29 to 5.31)
Hesdorffer 2009 [13] USA	Retrospective cohort study	Moderate	First episodes of acute symptomatic seizure due to central nervous system (CNS) infection, stroke, and traumatic brain injury (TBI)	Etiology of acute symptomatic seizure was stroke in 34.7%, TBI in 34.7%, and CNS infection in 30.6%. 55.9% male	First unprovoked seizure	Subsequent unprovoked seizure (seizure occurring at least 1 week after the etiologies)	10 years	First unprovoked seizure group had a significantly higher risk of subsequent unprovoked seizure (64.8%, 95% CI = 55.1–74.4%) compared to the first acute symptomatic seizure group (18.7%, 95% CI = 13.7–25.4%, *p* < 0.001)
Bedford 2001 [14] England	Prospective cohort study	High	Children who survived an episode of acute bacterial meningitis between 1985 and 1987	NA	Age- and sex-matched controls	Diagnosis of epilepsy	5 years	BM group = 116/1584 Control group = 37/1391
D’angio 1995 [15] USA	Retrospective cohort study	Moderate	100% Indian ancestry, < 5 years old at the time of diagnosis (bacterial meningitis group: *n* = 41, sibling controls *n* = 38, aged-matched controls *n* = 41)	Age at follow-up (years in mean; range in parentheses): 9.3 (1.2–18.5), sex: male, 69 (57.5%); female, 51 (42.5%)	Sibling controls and age and sex-matched controls	Diagnosis of epilepsy	3.6–15.0 years	BM group = 5/41, sibling controls = 0/38, 0/41
Stevens 2003 [16] England	Prospective cohort study	Moderate	Children aged 9–10 years old who had confirmed neonatal bacterial meningitis (up to 28 days of life) (bacterial meningitis group: *n* = 111 Hospital control group: *n* = 113 GP control group: *n* = 49)	Age at follow-up (years in mean; SD in parentheses): 9 (0.3), meningitis caused by group B streptococci, Gram negative bacteria (for example, *E. coli*) or Listeria monocytogenes. Bacterial meningitis defined as positive CSF culture	Hospital control group: controls matched for sex, birth date, birth weight (± 500 g), and hospital of birth GP control group: controls born at term and matched for sex and birth date	Diagnosis of epilepsy	9–10 years	BM group = 6/111, hospital controls = 2/113
Mohanty 2024 [17]	Retrospective cohort study	Low	3623	3623 individuals diagnosed with bacterial meningitis during childhood and 32 607 controls from the general population (median age at diagnosis, 1.5 [IQR, 0.4–6.2] years; 44.2% female and 55.8% male	Diagnosis of epilepsy	23.7 [IQR, 12.2–30.4] years	BM: 249/3623 Controls: 398/32607 HR: 4.14(3.46–4.96)	Mohanty 2024
Edmond 2010 [18]	Systematic review	Low	18,183 survivors of ABM (median number of survivors per study, 85 [IQR 47–148])	Hib (35·5%), pneumococcus (19·6%), meningococcus (16·4%), and other pathogens (12·0%) Median age at meningitis episode was 29 months (IQR 13–67 months), and 106 (79%) papers reported sequelae risk in children aged under 5 years 53 (40%) papers were from the European region	seizures	24 months (IQR 6–63 months)	12·6%	Edmond 2010
Adults								
Zelano 2020 [19] Sweden	Retrospective cohort study	High	Patients aged > 18 years who had inpatient hospital care for bacterial meningitis (ICD-10 code: A390, G00, G01, A17) and had survived 30 days after diagnosis/admission (index date). Participants were identified from the National Patient Register (NPR) (*n* = 39,040)		Age- and sex-matched controls who did not have brain infection	Diagnosis of epilepsy	Up to 17 years	BM group = 118/2812, hospital controls = 495/36228

## Discussion

### Summary of results

This systematic review aimed to evaluate the optimal duration of ASM in people with ASS due to acute meningitis. The search resulted in the inclusion of two studies: one randomized controlled trial (RCT) and one observational cohort study. Both studies provided indirect evidence as they included people with acute encephalitis syndrome and other neurological conditions in addition to bacterial meningitis and meningoencephalitis. The pooled analysis demonstrated no significant difference in seizure recurrence between earlier cessation (within 3 months) and late cessation (beyond 3 months) of ASM. However, the overall certainty of the evidence was rated as very low due to concerns related to indirectness and imprecision. Only one study evaluated adverse events, which were not reported in either group.

An additional important consideration from the Annegers et al. study [11] is that the absolute risk of repeat seizures at 1 year after meningitis was less than 5%, a finding important to be considered when physicians evaluate the risks and benefits of prolonged ASM in their patients. The harms of exposure to prolonged ASM may be assessed in view of this magnitude of risk of repeat seizure.

One included study was a cohort study, but provided a within-study comparison of shorter versus longer ASM duration and thus contributed a relative effect estimate relevant to our prespecified question. Although the included studies in the meta-analysis differed in age groups and underlying neurological diagnoses, since heterogeneity in study populations does not, in itself, warrant downgrading or subgroup analysis unless accompanied by inconsistency in results, this did not prompt any subgroup exploration [17, 18].

### Implications for practice

The findings of this review indicate that there is no clear evidence to support earlier or later cessation of ASM in people with ASS from acute meningitis. Given the very low certainty of evidence and the fact that the majority of people included in the studies had conditions other than bacterial meningitis, clinicians should exercise caution when extrapolating these results to their practice. Currently, the decision on the duration of ASM should be individualized, taking into consideration the clinical presentation, risk factors for seizure recurrence, and potential adverse effects of prolonged ASM use.

The lack of reported adverse events in one of the included studies suggests that short-term use of ASM may not lead to significant adverse effects, although this finding must be interpreted with caution due to the limited data available. Clinicians should also remain vigilant about the potential long-term consequences of seizure recurrence if ASM is discontinued prematurely.

Due consideration must be given to the complexities surrounding the decision to discontinue ASMs in people who have achieved seizure freedom. The core challenge lies in the uncertainty of whether seizure freedom is due to ongoing medication or if epilepsy remission has occurred [19]. Lamberink et al.’s meta-analysis [20] provides clinicians with predictive nomograms to assess the risk of seizure recurrence after ASM withdrawal, emphasizing that decisions should be individualized rather than based on a universal timeline of seizure freedom.

Historically, ASMs were discontinued after 1–2 years of seizure freedom due to the risk of side effects such as cognitive impairment and long-term consequences like osteopenia. However, newer ASMs have fewer adverse effects, complicating the risk–benefit analysis of continued drug use. It is reported that for every eight people who discontinue ASMs, one is likely to experience a seizure relapse, and some may develop treatment-resistant epilepsy, making it crucial to weigh the risks of withdrawal carefully [19].

This underscores the need for personalized discussions with patients, factoring in individual risks and benefits, rather than adhering to rigid protocols. While the nomograms offer guidance, patient and carer involvement in decision-making is essential to balance the potential recurrence risk against the benefits of stopping medication.

### Comparison to other studies

Our results align with the limited literature on the topic of ASM duration in ASS due to meningitis. A prior study by Zoons et al. [21] explored seizure outcomes in adults with bacterial meningitis and found a similarly low recurrence rate of seizures after ASM discontinuation. However, this study did not specifically compare earlier versus later ASM cessation, leaving a gap in understanding the optimal duration of treatment. Similarly, other studies examining ASM duration in people with central nervous system infections such as encephalitis have also reported conflicting results, with some studies suggesting that early cessation may increase the risk of recurrent seizures, while others found no significant differences in seizure recurrence based on ASM duration [22–26].

Thussu et al. [27] explored seizure recurrence in patients with single small enhancing CT lesions (SSECTL) who were treated with short—(6 months) versus long-duration (2 years) ASM. Recurrence was observed in 17% of patients in the short-duration group compared to 11.5% in the long-duration group. Verma et al. [28] provided similar findings in a cohort of patients with cerebral cysticercus granuloma. Recurrence of seizures occurred in 3.1% of patients treated with AEDs for 6 months and 4.8% of those treated for 2 years.

The wide variation in populations, etiologies, and treatment protocols across studies further complicates the ability to draw definitive conclusions. Our findings emphasize the need for more focused research specifically targeting patients with acute bacterial meningitis and encephalitis to clarify the role of ASM duration in these populations.

### Implications for research

There are substantial gaps in the current evidence base, particularly regarding the management of ASS in people with acute bacterial meningitis. Future research should focus on randomized controlled trials that specifically include this population. Additionally, standardized outcome measures, such as seizure recurrence rates, neurological outcomes, mortality, and adverse effects, are essential to allow for more robust comparisons across studies.

Subgroup analyses based on factors such as age, severity of meningitis, and underlying comorbidities could help to identify patient populations that may benefit from either early or late ASM cessation. Furthermore, the absence of data on the cost-effectiveness of early versus late ASM discontinuation warrants investigation, particularly in resource-limited settings where prolonged treatment may place a significant burden on healthcare systems.

### Recommendations

Based on the current evidence, we suggest that clinicians continue to individualize decisions regarding ASM duration in people with acute bacterial meningitis who have experienced ASS. Given the very low certainty of evidence supporting early ASM cessation, it may be prudent to adopt a more conservative approach, with close monitoring for seizure recurrence and adverse effects if treatment is stopped early.

For future guidelines, we recommend the development of well-designed, multicenter RCTs focused on the optimal duration of ASM in bacterial meningitis and encephalitis. Such studies should aim to establish a clearer understanding of the balance between the benefits and potential risks of earlier versus later ASM cessation, with a focus on long-term neurological outcomes, cost-effectiveness, and patient quality of life.

## Conclusions

This systematic review and meta-analysis aimed to assess the optimal duration of ASM in people with ASS due to acute meningitis. Despite a comprehensive search, no direct evidence was found specifically in this population, a limitation of this study. The available studies primarily included people with encephalitis, limiting the direct applicability of the findings to acute meningitis. The pooled analysis did not demonstrate a significant difference in seizure recurrence between early (≤ 3 months) and late (> 3 months) cessation of ASM, but the certainty of evidence was very low due to indirectness and imprecision.

As a result, clinical decision-making should be individualized, considering patient-specific factors such as seizure recurrence risk, underlying neurological injury, and potential adverse effects of prolonged ASM use. While indirect evidence from encephalitis suggests that shorter treatment durations may be pursued, more targeted research in acute meningitis is necessary to confirm the effectiveness and safety of this approach.

## Supplementary Information

Additional file 1: Appendix.

## Data Availability

All data used in this systematic review were obtained from published studies. No new datasets were generated. Extracted data are available from the corresponding author on reasonable request.

## Abbreviations

ABM: Acute bacterial meningitis
AED: Antiepileptic drug
ASM: Anti-seizure medicines
ASS: Acute symptomatic seizures
BM: Bacterial meningitis
CNS: Central nervous system
GRADE: Grading of recommendations, assessment, development and evaluation
ICD: International Classification of Diseases
IQR: Interquartile range
MD: Mean difference
NA: Not applicable
OR: Odds ratio
PICO: Population, intervention, comparator, outcome
PRISMA: Preferred Reporting Items for Systematic Reviews and Meta-Analyses
RCT: Randomized controlled trial
ROB: Risk of bias
ROBINS-I: Risk of Bias in Non-randomized Studies of Interventions
RR: Risk ratio
SD: Standard deviation
SMD: Standardized mean difference
SOC: Standard of care
SSECTL: Single small enhancing computed tomography lesion
WHO: World Health Organization
