# Supplementary material for: Duration of anti-seizure medicines started for acute symptomatic seizures due to acute meningitis: a systematic review and meta-analysis
Source: BMC Med. 2026 Apr 22;24:341. doi: 10.1186/s12916-026-04840-w (PMC13235151; doi:10.1186/s12916-026-04840-w)
Supplement: Supplementary file 1 — Additional file 1: Appendix. [file 12916_2026_4840_MOESM1_ESM.docx]

# Appendix I: Search strategy used to identify primary studies

Database: OVID Medline Epub Ahead of Print, In-Process & Other Non-Indexed Citations, Ovid MEDLINE(R) Daily and Ovid MEDLINE(R) 1946 to Present

Search Strategy:

--------------------------------------------------------------------------------

1 exp Meningitis/ (59046)

2 meningit*.mp. (81222)

3 1 or 2 (92471)

4 exp Anticonvulsants/ (154895)

5 (antiepileptic* or anti-epileptic* or antiseizure or anti-seizure or anticonvuls* or anti-convuls*).mp. (85306)

6 (Acetazolamid* or Aedon or Aethosuximide or Alodorm or Amizepin* or Ant?lepsin or Anxirloc or Arem or Ativan or Atretol or Avugane or Baceca or Barbexaclon* or Beclamid* or Biston or Bomathal or Brivaracetam or Bromid* or Calepsin or Carbagen or Carbamazepen* or Carbamazepin* or Carbatrol or Carbazepin* or Carbelan or Carisbamat* or Castilium or Celontin or Cerebyx or Chlonazepam or Chloracon or C?lorepin or C?lormethiazole or Clarmyl or Cloazepam or Clobam* or Clobator or Clobazam or Clofritis or Clonazepam* or Clonex or Clonopin or Clopax or Clorazepate or Comfyde or Convulex or Dapaz or Dasuen or Delepsine or Depacon or Depak* or Depamide or Deproic or Desitin or Diacomit or Diamox or Diastat or Diazepam or Difenilhidantoin* or Dihydantoin or Dilantin or Dimethadione or Dimethyloxazolidinedione or Diphenin* or Diphenylan or Diphenylhydantoin* or Distraneurin or Divalpr* or Dormicum or Ecovia or Emeside or Epanutin or Epiject or Epilepax or Epilex or Epilim or Episenta or Epitol or Epival or Eptoin or Equanil or Equetro or Ergenyl or Erimin or Erlosamide or Eslicarbazepine or Estazolam or Ethadione or Ethosucci* or Ethosuxi* or Ethotoin or Ethylphenacemide or Etosuxi* or Euhypnos or Exalief or Excegran or Ezogabine or Fanatrex or Felbam* or Felbatol or Fenitoin* or Fenobarbit* or Fenytoin* or Finlepsin or Fosphenytoin or Frisium or Fycompa or Gabapentin* or Gabapetin* or Gabarone or Gabitril or Gabrene or Ganaxolone or Garene or Gralise or Grifoclobam or Halogabide or Halogenide or Harkoseride or Hibicon or Hydroxydiazepam or Hypnovel or Iktorivil or Inovelon or Insoma or Intensl or Karbamazepin or Karidium or Keppra or Klonopin or Kriadex or Lacosamid* or Lamict* or Lamitor or Lamitrin or Lamogine or Lamotrigin* or Lamotrine or Landsen or Levanxol or Levetiracetam* or Lexin or Liskantin or Loraz or Lorazepam* or Losigamon* or Lucium or Luminal or Lyrica or Magnesium sulfat* or Magnesium sulphat* or Mebaral or Medazepam or Mephenytoin or Mephobarbit* or Mephyltaletten or Meprobamate or Meprospan or Mesantoin or Mesuximide or Methazolamid$ or Methsuximide or Methylacetazolamide or Methyloxazepam or Methylphenobarbit* or Midazolam or Miltown or Mogadon or Mylepsinum or Mylproin or Mysoline or Mystan or Neogab or Neptazane or Nesdonal or Neurontin or Neurotop or Nimetazepam or Nitrados or Nitrazadon or Nitrazepam or Nobrium or Nocturne or Noiafren or Norkotral or Normison or Normitab or Nortem or Novo-Clopate or Nuctalon or Nupentin or Nydrane or OCBZ or Onfi or Orfiril or Orlept or Ormodon or Ospolot or Oxcarbamazepin* or Oxcarbazepin* or Oxydiazepam or Pacisyn or Paraldehyde or Paramethadione or Paxadorm or Paxam or Peganone or Penthiobarbital or Pentothal or Perampanel or Petinutin or Petril or Phemiton or Phenacemide or Pheneturide or Phenobarbit* or Phensuximide or Phenylethylbarbit* or Phenylethylmalonylurea or Phenytek or Phenytoin* or Planum or Posedrine or Potiga or Pregabalin or Primidone or Prodilantin or Progabide or Prominal or Pronervon or Propofol or Prosom or Prysoline or Ravotril or Remacemide or Remestan or Remnos or Resimatil or Restoril or Retigabine or Riluzole or Rilutek or Riv?tril or Rudotel or Rufinamide or Rusedal or "RWJ-333369" or Sabril or Seclar or Sederlona or Selenica or Seletracetam or Sentil or Sertan or Sibelium or Signopam or Sirtal or Sodipental or Somnite or Stavzor or Stazepin* or Stedesa or Stiripentol or Sulthiam* or Sultiam* or Talampanel or Taloxa or Tasedan or Tegret?l or Telesmin or Temaze or Temazep* or Temesta or Temtabs or Tenox or Teril or Thiomebumal or Thionembutal or Thiopent* or Tiagabin* or Tiletamine or Timonil or Tiobarbit* or Tipiram* or Topamax or Topiram* or Tranmep or Tranxene or Trapanal or Tridione or Trileptal or Trimethadione or Trobalt or Urbadan or Urban?l or Valance or Valcote or Valium or Valnoctamide or Valparin or Valpro* or Versed or Vigabatrin* or Vimpat or Visano or VPA or Xilep or "YKP 509" or Zalkote or Zarontin or Zebinix or Zonegran or Zonisamid*).tw. (288544)

7 or/4-6 (367603)

8 3 and 7 (777)

9 limit 8 to (case reports or comment or editorial or letter or "review") (375)

10 8 not 9 (402)

Embase (OVID)

Search Strategy:

--------------------------------------------------------------------------------

1 exp meningit*/

2 (meningit*) or (infectious meningit*)

3 1 or 2

4 exp anticonvulsive agent/

5 (antiepileptic* or anti-epileptic* or antiseizure or anti-seizure or anticonvuls* or anti-convuls*).mp.

6 (Acetazolamid* or Aedon or Aethosuximide or Alodorm or Amizepin* or Ant?lepsin or Anxirloc or Arem or Ativan or Atretol or Avugane or Baceca or Barbexaclon* or Beclamid* or Biston or Bomathal or Brivaracetam or Bromid* or Calepsin or Carbagen or Carbamazepen* or Carbamazepin* or Carbatrol or Carbazepin* or Carbelan or Carisbamat* or Castilium or Celontin or Cerebyx or Chlonazepam or Chloracon or C?lorepin or C?lormethiazole or Clarmyl or Cloazepam or Clobam* or Clobator or Clobazam or Clofritis or Clonazepam* or Clonex or Clonopin or Clopax or Clorazepate or Comfyde or Convulex or Dapaz or Dasuen or Delepsine or Depacon or Depak* or Depamide or Deproic or Desitin or Diacomit or Diamox or Diastat or Diazepam or Difenilhidantoin* or Dihydantoin or Dilantin or Dimethadione or Dimethyloxazolidinedione or Diphenin* or Diphenylan or Diphenylhydantoin* or Distraneurin or Divalpr* or Dormicum or Ecovia or Emeside or Epanutin or Epiject or Epilepax or Epilex or Epilim or Episenta or Epitol or Epival or Eptoin or Equanil or Equetro or Ergenyl or Erimin or Erlosamide or Eslicarbazepine or Estazolam or Ethadione or Ethosucci* or Ethosuxi* or Ethotoin or Ethylphenacemide or Etosuxi* or Euhypnos or Exalief or Excegran or Ezogabine or Fanatrex or Felbam* or Felbatol or Fenitoin* or Fenobarbit* or Fenytoin* or Finlepsin or Fosphenytoin or Frisium or Fycompa or Gabapentin* or Gabapetin* or Gabarone or Gabitril or Gabrene or Ganaxolone or Garene or Gralise or Grifoclobam or Halogabide or Halogenide or Harkoseride or Hibicon or Hydroxydiazepam or Hypnovel or Iktorivil or Inovelon or Insoma or Intensl or Karbamazepin or Karidium or Keppra or Klonopin or Kriadex or Lacosamid* or Lamict* or Lamitor or Lamitrin or Lamogine or Lamotrigin* or Lamotrine or Landsen or Levanxol or Levetiracetam* or Lexin or Liskantin or Loraz or Lorazepam* or Losigamon* or Lucium or Luminal or Lyrica or Magnesium sulfat* or Magnesium sulphat* or Mebaral or Medazepam or Mephenytoin or Mephobarbit* or Mephyltaletten or Meprobamate or Meprospan or Mesantoin or Mesuximide or Methazolamid$ or Methsuximide or Methylacetazolamide or Methyloxazepam or Methylphenobarbit* or Midazolam or Miltown or Mogadon or Mylepsinum or Mylproin or Mysoline or Mystan or Neogab or Neptazane or Nesdonal or Neurontin or Neurotop or Nimetazepam or Nitrados or Nitrazadon or Nitrazepam or Nobrium or Nocturne or Noiafren or Norkotral or Normison or Normitab or Nortem or Novo-Clopate or Nuctalon or Nupentin or Nydrane or OCBZ or Onfi or Orfiril or Orlept or Ormodon or Ospolot or Oxcarbamazepin* or Oxcarbazepin* or Oxydiazepam or Pacisyn or Paraldehyde or Paramethadione or Paxadorm or Paxam or Peganone or Penthiobarbital or Pentothal or Perampanel or Petinutin or Petril or Phemiton or Phenacemide or Pheneturide or Phenobarbit* or Phensuximide or Phenylethylbarbit* or Phenylethylmalonylurea or Phenytek or Phenytoin* or Planum or Posedrine or Potiga or Pregabalin or Primidone or Prodilantin or Progabide or Prominal or Pronervon or Propofol or Prosom or Prysoline or Ravotril or Remacemide or Remestan or Remnos or Resimatil or Restoril or Retigabine or Riluzole or Rilutek or Riv?tril or Rudotel or Rufinamide or Rusedal or "RWJ-333369" or Sabril or Seclar or Sederlona or Selenica or Seletracetam or Sentil or Sertan or Sibelium or Signopam or Sirtal or Sodipental or Somnite or Stavzor or Stazepin* or Stedesa or Stiripentol or Sulthiam* or Sultiam* or Talampanel or Taloxa or Tasedan or Tegret?l or Telesmin or Temaze or Temazep* or Temesta or Temtabs or Tenox or Teril or Thiomebumal or Thionembutal or Thiopent* or Tiagabin* or Tiletamine or Timonil or Tiobarbit* or Tipiram* or Topamax or Topiram* or Tranmep or Tranxene or Trapanal or Tridione or Trileptal or Trimethadione or Trobalt or Urbadan or Urban?l or Valance or Valcote or Valium or Valnoctamide or Valparin or Valpro* or Versed or Vigabatrin* or Vimpat or Visano or Xilep or Zalkote or Zarontin or Zebinix or Zonegran or Zonisamid*).tw.

7 or/4-6

8 3 and 7

9 (rat or rats or mouse or mice or swine or porcine or murine or sheep or lambs or pigs or piglets or rabbit or rabbits or cat or cats or dog or dogs or cattle or bovine or monkey or monkeys or trout or marmoset$1).ti. and animal experiment/

10 Animal experiment/ not (human experiment/ or human/)

11 9 or 10

12 8 not 11

Cochrane Library (Wiley)

Comment:

ID Search Hits

#1 MeSH descriptor: [Meningit*] explode all trees

#2 (meningit*)

#3 #1 or #2

#4 MeSH descriptor: [Anticonvulsants] explode all trees

#5 (antiepileptic* or anti-epileptic* or antiseizure or anti-seizure or anticonvuls* or anti-convuls*):ti,ab,kw (Word variations have been searched)

#6 (Acetazolamid* or Aedon or Aethosuximide or Alodorm or Amizepin* or Ant?lepsin or Anxirloc or Arem or Ativan or Atretol or Avugane or Baceca or Barbexaclon* or Beclamid* or Biston or Bomathal or Brivaracetam or Bromid* or Calepsin or Carbagen or Carbamazepen* or Carbamazepin* or Carbatrol or Carbazepin* or Carbelan or Carisbamat* or Castilium or Celontin or Cerebyx or Chlonazepam or Chloracon or C?lorepin or C?lormethiazole or Clarmyl or Cloazepam or Clobam* or Clobator or Clobazam or Clofritis or Clonazepam* or Clonex or Clonopin or Clopax or Clorazepate or Comfyde or Convulex or Dapaz or Dasuen or Delepsine or Depacon or Depak* or Depamide or Deproic or Desitin or Diacomit or Diamox or Diastat or Diazepam or Difenilhidantoin* or Dihydantoin or Dilantin or Dimethadione or Dimethyloxazolidinedione or Diphenin* or Diphenylan or Diphenylhydantoin* or Distraneurin or Divalpr* or Dormicum or Ecovia or Emeside or Epanutin or Epiject or Epilepax or Epilex or Epilim or Episenta or Epitol or Epival or Eptoin or Equanil or Equetro or Ergenyl or Erimin or Erlosamide or Eslicarbazepine or Estazolam or Ethadione or Ethosucci* or Ethosuxi* or Ethotoin or Ethylphenacemide or Etosuxi* or Euhypnos or Exalief or Excegran or Ezogabine or Fanatrex or Felbam* or Felbatol or Fenitoin* or Fenobarbit* or Fenytoin* or Finlepsin or Fosphenytoin or Frisium or Fycompa or Gabapentin* or Gabapetin* or Gabarone or Gabitril or Gabrene or Ganaxolone or Garene or Gralise or Grifoclobam or Halogabide or Halogenide or Harkoseride or Hibicon or Hydroxydiazepam or Hypnovel or Iktorivil or Inovelon or Insoma or Intensl or Karbamazepin or Karidium or Keppra or Klonopin or Kriadex or Lacosamid* or Lamict* or Lamitor or Lamitrin or Lamogine or Lamotrigin* or Lamotrine or Landsen or Levanxol or Levetiracetam* or Lexin or Liskantin or Loraz or Lorazepam* or Losigamon* or Lucium or Luminal or Lyrica or Magnesium sulfat* or Magnesium sulphat* or Mebaral or Medazepam or Mephenytoin or Mephobarbit* or Mephyltaletten or Meprobamate or Meprospan or Mesantoin or Mesuximide or Methazolamid$ or Methsuximide or Methylacetazolamide or Methyloxazepam or Methylphenobarbit* or Midazolam or Miltown or Mogadon or Mylepsinum or Mylproin or Mysoline or Mystan or Neogab or Neptazane or Nesdonal or Neurontin or Neurotop or Nimetazepam or Nitrados or Nitrazadon or Nitrazepam or Nobrium or Nocturne or Noiafren or Norkotral or Normison or Normitab or Nortem or Novo-Clopate or Nuctalon or Nupentin or Nydrane or OCBZ or Onfi or Orfiril or Orlept or Ormodon or Ospolot or Oxcarbamazepin* or Oxcarbazepin* or Oxydiazepam or Pacisyn or Paraldehyde or Paramethadione or Paxadorm or Paxam or Peganone or Penthiobarbital or Pentothal or Perampanel or Petinutin or Petril or Phemiton or Phenacemide or Pheneturide or Phenobarbit* or Phensuximide or Phenylethylbarbit* or Phenylethylmalonylurea or Phenytek or Phenytoin* or Planum or Posedrine or Potiga or Pregabalin or Primidone or Prodilantin or Progabide or Prominal or Pronervon or Propofol or Prosom or Prysoline or Ravotril or Remacemide or Remestan or Remnos or Resimatil or Restoril or Retigabine or Riluzole or Rilutek or Riv?tril or Rudotel or Rufinamide or Rusedal or Sabril or Seclar or Sederlona or Selenica or Seletracetam or Sentil or Sertan or Sibelium or Signopam or Sirtal or Sodipental or Somnite or Stavzor or Stazepin* or Stedesa or Stiripentol or Sulthiam* or Sultiam* or Talampanel or Taloxa or Tasedan or Tegret?l or Telesmin or Temaze or Temazep* or Temesta or Temtabs or Tenox or Teril or Thiomebumal or Thionembutal or Thiopent* or Tiagabin* or Tiletamine or Timonil or Tiobarbit* or Tipiram* or Topamax or Topiram* or Tranmep or Tranxene or Trapanal or Tridione or Trileptal or Trimethadione or Trobalt or Urbadan or Urban?l or Valance or Valcote or Valium or Valnoctamide or Valparin or Valpro* or Versed or Vigabatrin* or Vimpat or Visano or VPA or Xilep or Zalkote or Zarontin or Zebinix or Zonegran or Zonisamid*):ti,ab,kw (Word variations have been searched)

#7 #4 or #5 or #6

#8 #3 and #7 in Trials

Appendix II: Risk of bias assessment of included studies

A) Risk of bias in the included RCT using ROB2.0 tool

| **Trial and outcome name** | **1) Bias arising from the randomization process** | **Domain 1 justification** | **2) Bias due to deviations from the intended intervention** | **Domain 2 justification** | **3) Bias due to missing outcome data** | **Domain 3 justification** | **4) Bias in measurement of the outcome** | **Domain 4 justification** | **5) Bias in selection of the reported results** | **Domain 5 justification** | **6) Other biases (e.g., competing risks)** | **Domain 6 justification** |
| --- | --- | --- | --- | --- | --- | --- | --- | --- | --- | --- | --- | --- |
| Dhawan S R, 2021, India | Low | Randomization by computer generated, allocation concealed, baseline characteristics seems simillar | Probably high | Open label | Low | No significant loss to follow up | Low | Outcome assessor blinded | Low | Same as published protocol | Probably low |  |

B) Risk of bias in included cohort studies using ROBINS-I

| Outcome | Study (Author Last Name & Year) | Adjusted /Unadjusted Analysis | ROBINS-I Assessment | Confounding Bias | Selection Bias | Classification Bias | Bias from Deviations from Intended Intervention | Missing Data Bias | Measurement Bias | Selective Reporting Bias |
| --- | --- | --- | --- | --- | --- | --- | --- | --- | --- | --- |
| Seizure recurrence | Herzig‐Nichtweiß 2023 | A | Low | Low | High | Low | Low | Low | Low | Low |
